# Item memorability has no influence on value-based decisions

**DOI:** 10.1038/s41598-022-26333-5

**Published:** 2022-12-21

**Authors:** Xinyue Li, Wilma A. Bainbridge, Akram Bakkour

**Affiliations:** 1 Department of Psychology, University of Chicago, 5848 S University Ave, Chicago, IL 60637 USA; 2 Neuroscience Institute, University of Chicago, 5812 S Ellis Ave, Chicago, IL 60637 USA

**Keywords:** Psychology, Human behaviour

## Abstract

While making decisions, we often rely on past experiences to guide our choices. However, not all experiences are remembered equally well, and some elements of an experience are more memorable than others. Thus, the intrinsic memorability of past experiences may bias our decisions. Here, we hypothesized that individuals would tend to choose more memorable options than less memorable ones. We investigated the effect of item memorability on choice in two experiments. First, using food images, we found that the same items were consistently remembered, and others consistently forgotten, across participants. However, contrary to our hypothesis, we found that participants did not prefer or choose the more memorable over the less memorable items when choice options were matched for the individuals’ valuation of the items. Second, we replicated these findings in an alternate stimulus domain, using words that described the same food items. These findings suggest that stimulus memorability does not play a significant role in determining choice based on subjective value.

## Introduction

Decisions are ubiquitous in everyday life. When deliberating about what to eat, for example, an individual can compare the values they place on different choice options and choose the food that holds higher value. When one of the choice options holds much higher subjective value than the others, the choice is easy, and a decision is made quickly. However, when deciding between choice options of equal value, the decision is more difficult, and the same individual requires more time to make a decision^1–4^. Previous research has shown that some of this additional time for difficult decisions may go toward memory processes that help resolve the decision conflict^5,6^. The idea is that samples of evidence for different choice options are drawn from memory and integrated into value to guide decisions^7,8^. However, it is unlikely that memory processes in service of value-based decisions are deployed equally across choice options, given that not all items are equally memorable. Some items are intrinsically more memorable than others across observers^9,10^. If you were asked to remember whether you’ve seen a particular image of a slice of pizza before when presented for the second time and later asked if you’ve seen a particular image of saltine crackers before when presented for the second time, which of the images of food do you think you would be more likely to accurately remember? We find in this study that an image of a slice of pizza is more memorable than an image of saltine crackers (see Fig. 2 below). But what makes some items more memorable than others? More memorable items tend to have more semantic connections and are neurally represented more similarly in brain regions specialized in processing late perceptual and memory information when compared to forgettable items^10,11^. This suggests that more memorable items may be embedded within a richer associative network in memory and may elicit more samples of evidence from memory that are input to a decision process when memorable items appear as options in a choice context. Thus, it stands to reason that more memorable appetitive choice options would have more influence on individuals’ decisions than forgettable appetitive options. The current study investigates the influence of item memorability on people’s value-based choices within a framework that places memory at the input of a decision process.

Memorability refers to the extent to which items are accurately remembered across people^9^. Despite individual differences in prior experiences and familiarity, people consistently remember and forget the same images^12^. Crucially, this implies that images can be quantified by a “memorability score” that reflects the likelihood that a given image will be remembered across people. A growing body of work has shown that the memorability of a given image is indeed constant across different experimental tasks and image contexts^13^, participant samples^10^, and image categories^14,15^. This consistency across tasks, people, and images occurs regardless of the specific measure used, including corrected recognition (CR), d-prime, hit rate, and false alarm rate^12,16^. Functionally, given that memorability is intrinsic to the stimulus, it can be quantified using trained machine learning algorithms and used to predict memory behavior^17–19^. Memorable items also result in stereotyped brain patterns in late visual and memory areas^11,16^, and are reinstated earlier in the brain and cause more memory intrusions than forgettable items^10^. These results suggest that more memorable items are prioritized for retrieval and their associations in memory may be sampled sooner during decisions when compared to forgettable items.

The ability to remember associations between elements of an event or a series of events (i.e., relational memory) is essential to many forms of decision making^6^. By retrieving experiences related to the choice options at hand, individuals can predict the outcome of different choices to guide their decision^8,20,21^. This important role for relational memory in choice is supported by past research that identified a role for the hippocampus (a brain region that is crucial for relational memory) in deliberation during value-based choices^5^. Healthy individuals deliberate more when the decision is difficult, like when the values of choice options under consideration are matched. However, amnesic patients due to damage to the hippocampus did not exhibit a relationship between deliberation time and choice difficulty. These findings provide evidence for a necessary role of the hippocampus in deliberation and suggest an important role for relational memory in normative value-based decision making processes. However, in that research, the memorability of choice options was not controlled for. Indeed, as mentioned previously, more memorable items may be prioritized for retrieval during decision making and bias choices.

Here, we investigated whether people’s decisions are influenced by the memorability of choice options. By bridging prior research on both value-based decision making and memorability, the current work clarifies the cognitive mechanisms underlying value-based decisions. Based on previous literature that implicates relational memory in biasing decision making^1,6^, we hypothesized that participants will be more likely to choose items that have a higher memorability score when the subjective values of the choice options are the same. According to our hypothesis, participants will need more time to deliberate to resolve conflict when memorability is matched across choice options. Thus, we predicted that there would be a relationship between reaction times and the difference in memorability scores of the choice options, controlling for value.

In the two experiments reported here, we tested the predicted relationship between memorability and value-based choices for food items. We chose to use commonly available and familiar food items as visual stimuli because food is an appetitive stimulus that individuals generally approach and form positive associations with. Furthermore, individuals are accustomed to making choices among food options and likely have had a number of prior experiences with these foods that they can draw on when deliberating their choice. Our hypothesis states that more memorable food images would more readily elicit retrieval of participants’ experiences associated with those appetitive food items, providing more samples of positive evidence to a decision process in favor of memorable foods and consequently biasing choices in favor of those items over less memorable foods. We examined the relationship between memorability and choice across two different modalities, testing visual-based decisions (with food photographs) and semantic-based decisions (with food names). In Experiment 1, we first measured the memorability scores of food images using a continuous recognition memory task (Experiment 1A). We then tested another group of participants’ value-based choices for these foods (Experiment 1B). In a second experiment, we replicated our results using names of the food items depicted in the images used in Experiment 1.

## Experiment 1

The goal of Experiment 1 was to (1) measure memorability scores of images of food through a “memory game” (Isola et al., 2011, Experiment 1A), and (2) test whether participants tended to choose more memorable food items using a value-based choice task (Experiment 1B).

### Experiment 1A: memorability task

#### Methods

##### Stimuli

The image stimuli in Experiment 1 were taken from the Food Folio stimulus set, which is publicly available for download and published by the Columbia Center for Eating Disorders^22^. This stimulus set contains 138 images of food items that are familiar to the U.S. population and form a variety of categories (high/low calories and fat, fruits, snacks, etc.) All images were 410 × 410 pixels in size and depicted different food items located at the center of the image placed on a white plate with a black background.

##### Participants

Two hundred and nineteen participants in total were recruited on Amazon Mechanical Turk (AMT) and compensated $7.50/h for their time. All participants provided informed consent before participating in the study. Instructions for the task were provided to the participants at the beginning of the experiment, followed by two comprehension questions regarding the instructions. The comprehension checks were designed to ensure participants were proficient in English and fully understood the task. Only participants who answered the questions correctly within 3 attempts continued to the task. Twenty participants who performed poorly on the memory task (defined by fewer than 3 hits or 3 correct rejections) were excluded. This resulted in a final sample of 199 participants (35% women, ages ranging between 18 and 35). The sample size was selected so that each image was viewed as a target in the memory task by at least 40 participants, to allow for a reliable estimate of image memorability^14^. All experimental procedures were approved by the University of Chicago Internal Review Board (IRB) and the studies were conducted according to approved procedures. The research was performed according to the guidelines and regulations laid out by the IRB.

##### Task

**Figure 1:**
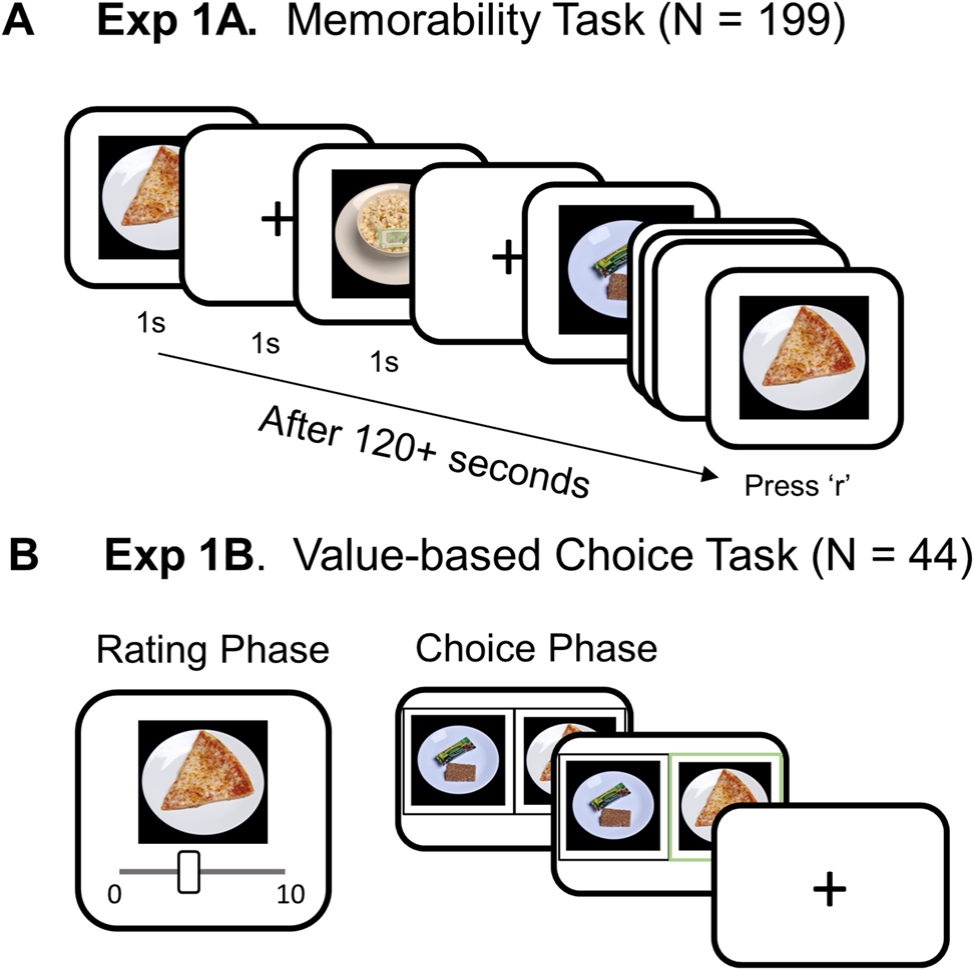
Diagram of tasks in Experiment 1. (**A**) Memorability task (Experiment 1A). Participants (N = 199) were asked to view a stream of images and press the 'r' key on the keyboard if they saw a repeated image. Images were randomly classified as filler or target for each participant, and the spacing between the first and second display was different for targets and fillers. (**B**) Value-based choice task (Experiment 1B). In the rating phase, participants (N = 44) were asked to rate all of the 138 food items based on their preference (0 = least prefer to eat this food, 10 = most prefer to eat this food). In the choice phase, participants were asked to choose the item that they would like to eat as a snack. Participants had 3 s to make their choice. The chosen item was then highlighted for 500 ms. Choice trials were separated by 1000 ms intertrial interval. The photographs used in this figure are publicly available for download at https://osf.io/483mx^22^.

TO measure each food item's memorability score, participants engaged in a continuous recognition task where they identified repeats amongst a stream of food images. We adopted and modified the Memorability Experiment Maker to create our online experiment^23^. During the study, participants viewed a stream of food images and were asked to press the ‘r’ key on the keyboard when they saw an image for a second time^12^. Each image was displayed for 1 s, with a 1-s interstimulus interval (Fig. 1A). For each participant, the stimuli were randomly divided into 29 targets and 109 fillers. The targets were repeated after more than 60 trials (120 s). The fillers were used to fill in the gaps randomly between the first and second presentations of targets, and some of them repeated at most once after a brief delay (1–7 trials). The task took approximately 14 min to complete. Only the performance for target images was used to calculate the memorability score. Filler responses were used to check if participants were paying attention to and performing the task as instructed. Each image was randomly assigned as a target for 42 participants on average. After completing the task, participants provided demographic information and answered questions about their general food preferences (i.e., whether they are vegetarian, and whether they have any food allergies).

##### Data analysis

Analyses of the data were conducted using R software^24^. The memorability score of each food item was calculated as Corrected Recognition (CR), which is the hit rate minus the false alarm rate for trials where the image was assigned as the target^9^.

##### Consistency of memorability scores

To evaluate whether the memorability scores were consistent across participants, we split participants into two random halves and calculated memorability scores (CR) for all images for each half. We repeated this process for 1000 iterations. We then calculated the Spearman rank correlation between the two sets of CR scores (first random half versus second random half) across items for each iteration.

##### Data and code availability

Data and analysis code for all experiments is available at the following link: https://github.com/christineli0330/mem_dm_share.

#### Results

**Figure 2:**
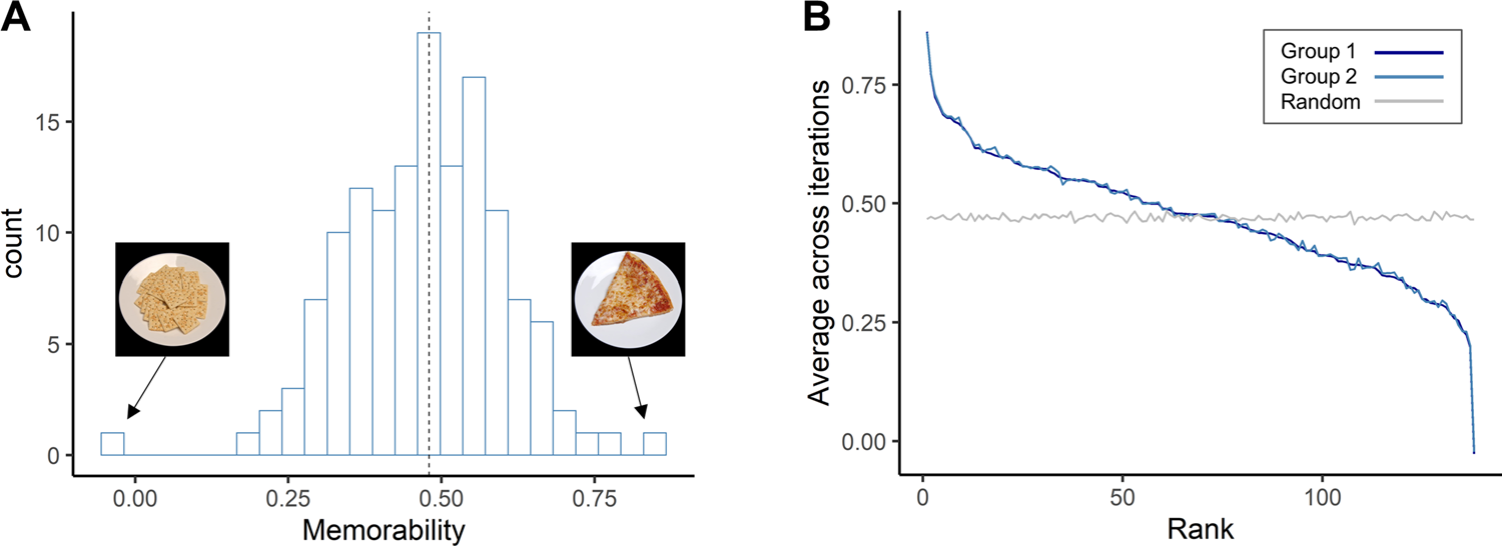
Image memorability scores differ across items but are consistent across participants. (**A**) Distribution of calculated memorability scores (corrected recognition, CR) for food items with an example of items with extreme memorability scores. The median of the memorability scores is 0.48 (dotted vertical line). (**B**) Consistency of image memorability scores. Split-half consistency is depicted using ranked average memorability scores across 1000 iterations of random sample splits. For each iteration, we randomly grouped participants into 2 groups: Group 1 and Group 2, and calculated the memorability scores for each image. We plotted the image memorability scores for Group 1 participants (dark blue line) against their memorability rank on the x-axis, the image memorability scores for Group 2 participants (light blue line), and the line for a permuted estimate of chance (grey line) against the same memorability rank. Group 1 and Group 2 show significant consistency in which images they remembered and forgot (*p* < 0.001). The photographs used in this figure are publicly available for download at https://osf.io/483mx^22^.

The calculated memorability scores showed variability across 138 food stimuli with a median of 0.48 (Fig. 2A). The minimum score was -0.03 for saltine crackers, meaning that participants did poorly in remembering this image and tended to misidentify it as old when seeing it for the first time. The maximum score was 0.86 for sliced pizza, meaning that participants were good at remembering this image and correctly identifying it with minimal false alarms. The consistency analysis revealed that the memorability scores were consistent across participants (*ρ* = 0.29, *p* < 0.001, Fig. 2B), indicating that participants indeed tended to remember and forget the same images, regardless of individual experiences. We also predicted the memorability scores for images using ResMem, which is a recently developed deep neural network (DNN) model for predicting image memorability (hit rate) across a wide range of images^19^. ResMem predictions were significantly positively correlated with the hit rates measured in Experiment 1A (*r(136)* = 0.24, *p* = 0.004), suggesting that the memorability scores are robust and intrinsic to the stimuli. The consistency of memorability across participants and methods implies that memorability is a meaningful property of these images: some of these food images are easier to remember than others. This allows us to directly test how images with different memory fates influence decision making.

### Experiment 1B: value-based choice task

#### Methods

##### Stimuli

The stimuli were the same as in Experiment 1A.

##### Participants

Sixty-one participants were recruited on the online behavioral research platform CloudResearch^25^, where the experiment was conducted through AMT, and participants were compensated $7.50/h for their time. All participants provided informed consent before participating in the study. Seventeen participants were excluded because they failed to pass the comprehension check or did not reach inclusion criteria based on their reaction times (RT) and choice performance. The criteria are detailed in the next paragraph. This resulted in a final sample of 44 participants (30% women, ages ranging between 21 and 42). All participants in the two experiments were located in the U.S. and proficient in English. All experimental procedures were approved by the University of Chicago IRB and conducted according to approved procedures. The research was performed according to the guidelines and regulations laid out by the IRB.

We established exclusion criteria to make sure participants fully understood our task and were attentive. Some participants met multiple exclusion criteria. First, we checked if participants tended to choose left or right items disproportionately. We excluded three participants whose probability of choosing items on the right was 2 standard deviations away from the mean of all participants, indicating that they almost always chose the item on the right side of the screen even though right/left placement of the more desirable item in a pair was randomized across trials. We also excluded 2 participants because their average RT was less than 300 ms in more than half of the trials, suggesting that they were not making value judgments on most trials and consistently ignored messages on the screen alerting them to the fact that they were making choices too quickly (see choice phase methods below). Moreover, we calculated delta value (Δvalue): the difference in subjective values of the two choice options. We z-scored the subjective values placed on all items for each participant, and then we used the z-scored value of the item on the right minus the z-scored value of the item on the left for each choice trial. Then, we performed logistic regressions to test the effect of Δvalue on choices for each participant separately. We excluded 12 participants that had negative parameter estimates or a non-significant effect of Δvalue on choices since this indicated that their rated subjective values (from the rating phase described below) were not in accord with their choice preferences. Thus, these 12 participants were either not veridical in their ratings, or in their choices, or both. Note that this means that, by necessity, we will observe a relationship between value and choice in the analyses of Δvalue. However, our key questions focus instead on the role of memorability in choice.

##### Task

Experiment 1B was made up of two phases: a rating phase and a choice phase (Fig. 1B).

##### Rating phase

Participants were asked to rate all 138 food stimuli on a scale from 0 to 10 for how much they would prefer to eat that food today. A score of 0 indicated they did not want to eat that food at all, while 10 indicated that they most strongly preferred to eat that food. The images were presented on the screen one at a time, with a slider bar at the bottom ranging from 0 (least) to 10 (most). Participants used a computer mouse to make their responses. Preference ratings that reflected the subjective value placed on the food stimuli (from 0 to 10) were recorded.

##### Choice phase

After the rating phase, the 138 food items were ranked based on the individual participant’s subjective value ratings that they just reported during the rating phase. The food items were then grouped into two sets of pairs for the choice phase of the experiment. First, items were rank ordered based on subjective value (obtained in the rating phase) and items with adjacent ranks (item with rank order 1 + item with rank order 2, item with rank order 3 + item with rank order 4, etc.) were paired together, forming 69 pairs of items of similar value. Second, items that were distant in terms of subjective value were paired together, with a distance of 68 ranks (e.g. the 1st ranked and the 69th ranked items were paired together), forming another 69 pairs of items. Therefore, half of the image pairs were very close in terms of subjective value, while half were distant in terms of subjective value. The combined 138 pairs of items were unique for each participant, and all pairs were presented once in separate trials during the choice phase.

After a short break following the rating phase, participants took part in the choice task. In the choice phase, two food items appeared on the screen, placed to the left and right of a central fixation point. Participants were instructed to choose one item per trial by pressing one of two keys on their keyboard; they pressed the ‘j’ key to select the item on the left and the ‘k’ key to select the item on the right. They were allotted up to 3 s to make their choice. After they made a response, the chosen item was highlighted with a green box for 0.5 s, followed by fixation. The trial duration was fixed at 3.5 s so that participants could not finish the task early by making faster choices. If they made the choice faster than 3 s, the rest of the time was added to the inter-trial fixation period. Participants were notified if they made a choice too quickly (shorter than 300 ms) or too slowly (longer than 3 s). The order of trials and the positions of the items on the screen (left/right) were randomized. Participants’ choices and RT were recorded.

##### Data analysis

We calculated Δmem for each trial. First, memorability scores (which are the corrected recognition scores for all items) were z-scored across items. Then Δmem was calculated as z-scored memorability for the item on the right side of the screen minus the z-scored memorability score for the item on the left side of the screen. We used the lme4 package in R^26^ and performed repeated-measures mixed-effects logistic regression models to test the effects of |Δvalue| and |Δmem| on value-based choices among all participants separately. Significance was determined by calculating p-values based on likelihood ratio tests. In order to investigate the relationship between value and choice behavior controlling for any influence of memorability, we used a median split on |Δmem| to select the half of the choice trials where items had a low difference in memorability. We entered |Δvalue| as a fixed effect in the mixed-effects logistic regression model to predict the probability of choosing the higher-valued item. As random effects, we included intercepts for subjects and by-subject random slopes for the effect of |Δvalue|. For a second model, we used a median split on |Δvalue| to select the half of the trials where items had a low value difference in order to investigate the relationship between memorability and choice behavior without influence from value. We entered |Δmem| as fixed and by-subject random slopes in the mixed-effects logistic regression model in predicting the probability of choosing more memorable items.

Beyond choice behavior, we also examined the relationship between RT during the choice phase and the two variables of |Δvalue| and |Δmem| by fitting a linear mixed-effects model. Prior studies have shown that participants tend to make slower decisions if the subjective values of the two items are similar^3–5^. Therefore, when the values of the two items in a pair are similar, we predicted slower RTs compared to when the two items differed on value. We also predicted that when items are close in terms of value but different in terms of memorability, participants would tend to spend longer on choices when |Δmem| is small compared to when |Δmem| is large. Reaction times are not normally distributed; thus we logistically transformed the RT data to reduce skewness and conform to the regression model’s assumptions. For testing the relationship between RT and value difference, we entered |Δvalue| as a fixed effect to predict the log-transformed RT in the linear mixed-effects model. We entered random intercepts for subjects and by-subject random slopes for the random effect of |Δvalue|. Also, to explore the relationship between RT and the difference in memorability between options, we entered |Δmem| as a fixed effect to predict the log-transformed RT in the linear mixed-effects model. We also entered random intercepts for subjects and by-subject random slopes for the random effect of |Δmem|.

To explore the relationship between the subjective values obtained in the rating phase and the memorability scores, we averaged subjective value ratings across participants for each of the 138 items, and calculated the correlation between averaged subjective ratings and memorability scores.

#### Results

##### Relationship between value and choice

**Figure 3:**
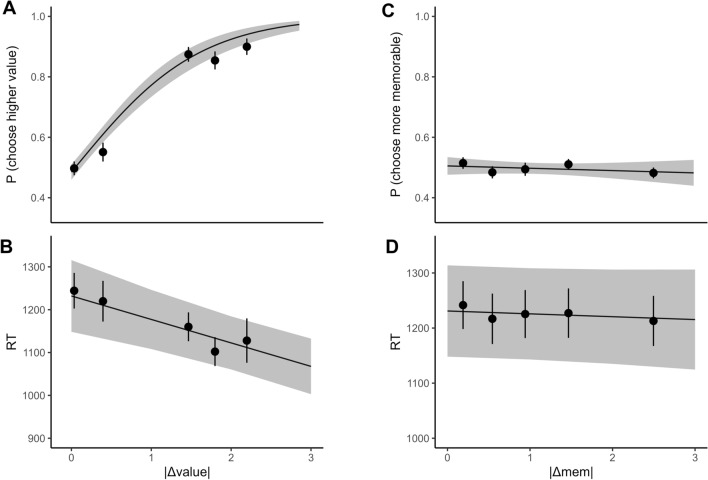
Subjective value, but not image memorability relates to choice behavior and speed. The proportion of higher-valued-item preferences (**A**) and RT (**B**) plotted as a function of |Δvalue| for pairs of items that have low |Δmem|. The proportion of higher-memorability choices (**C**) and RT (**D**) plotted as a function of |Δmem| for pairs of items that have low |Δvalue|. All of the models had 2939 trials (the trials in panel A and B are the same—low |Δmem| trials—but different in panels C and D, which were low |Δvalue| trials). In each plot, the solid black line represents the predicted regression line. The shaded gray area represents the 95% confidence intervals. The trial data were binned into 5 equal-sized bins. The black dots indicate the mean proportion of choosing a higher-valued item (top-left), choosing the more memorable item (top-right), or mean RT (bottom) across participants within bins. Error bars are standard error of the mean across participants within bins.

First, we tested the relationship between choices and item value. To eliminate the potential influence of memorability on choices, we selected trials where |Δmem| was low or close to zero using a median split. The mixed-effects logistic regression showed that |Δvalue| was related to choice behavior (*Odds Ratio (OR)* = 3.60, 95% *Confidence Interval (CI)* = 2.90−4.46, *p* < 0.001), meaning that the difference between the values significantly predicted participants’ choice of items in trials for which |Δmem| was near zero (Fig. 3A). Note that based on our exclusion criteria, this relationship is necessarily significant. Furthermore, in the linear mixed-effects model of RT, |Δvalue| significantly negatively related to RT (Fig. 3B), where the greater the difference in value between the two items, the less time was needed to make the decision (*β* = − 0.04, 95% *CI* = − 0.06 to − 0.02, *p* < 0.001). This finding is in accord with previous studies that showed individuals tend to make faster choices for larger |Δvalue| pairs^3–5^. The effects of Δvalue on choice and RT remain significant when entered in a regression model that includes Δmem as a regressor (see supplementary information), suggesting that much variance in choices and RT loads on Δvalue.

##### Relationship between memorability and choice

To answer the main question posed in this study, we also examined the effect of |Δmem| on participants’ choices and RTs. We selected trials where |Δvalue| was low or close to zero using a median split (Supplement S1) so that the effect of value difference can be controlled. Contrary to our prediction, |Δmem| was not related to choice behavior (*OR* = 0.97, 95% *CI* = 0.89–1.05,* p* = 0.47, Fig. 3C). The upper end of the 95% confidence interval for the effect of |Δmem| on the choice of more memorable items is well below 1.68, which is considered a small effect size equivalent to a Cohen’s d of 0.2^27^. We were well-powered to detect a small effect size of OR = 1.68 with 44 participants (see supplementary information). Post hoc power analysis for the observed effect size at a range of simulated sample sizes revealed that simulated power never rose above 6% for up to a simulated sample size of 500 (Fig. S1). Following recommendations by Lakens and colleagues for determining the informativeness of null results^28^, we calculated a Bayes Factor for our regression model. To accomplish this, we reran the same mixed-effects regression using a Bayesian fitting procedure (see supplementary information). We replicated the same effect size and found that there was no credible influence of |Δmem| on choices. More importantly Bayesian fitting allowed us to calculate a Bayes Factor (BF) for the model. Here, we report BF01, which, by convention provides evidence in favor of the null hypothesis. For the model that tested the effect of |Δmem| on choices, BF01 = $3.70\times {10}^{4}$, providing very strong evidence in favor of the null hypothesis that there was no effect of |Δmem| on choices. To ensure that our choice for the width of the priors in Bayesian model fitting did not affect our inference, we refit the model with different prior widths and calculated BF01s for these different widths of priors (Fig. S2). Even with quite narrow priors, BF01 provided strong evidence in favor of the null.

In addition, there was no significant relationship between |Δmem| and RT (*β* = − 0.004, 95% *CI* = − 0.02 to 0.01, *p* = 0.51, Fig. 3D). To calculate a Bayes Factor, we reran the regression in a Bayesian framework^29,30^ and replicated the effect size (see supplementary information). More importantly, for this model, BF01 = $1.40\times {10}^{6}$, providing overwhelming evidence in favor of the null hypothesis that there was no effect of |Δmem| on RT. Finally, we reran the choice and RT regressions including all participants (including those that met our exclusion criteria) and the results did not change (see supplementary information).

Rather than using measured memorability, we included the predictions of the deep neural network model ResMem^19^ for the food images in this study in our regression analyses (see supplementary information). We found that Δvalue related to choices and RT when accounting for the difference in ResMem predictions (ΔResMemPrediction). The regression parameter estimate for the effect of ΔResMemPrediction on choices indicates that participants significantly preferred items with lower ResMem predictions when accounting for Δvalue. ΔResMemPrediction did not relate to RT when accounting for Δvalue. There was also no effect of the interaction between Δvalue and ΔResMemPrediction on either choices or RTs.

We also reasoned that perhaps the sum of the memorability scores across the two items in a pair (SumMem), rather than Δmem, would have an effect on choices, but especially RT, since the amount of information in memory available to sample in service of a value based decision may be low in the case of a choice between two forgettable items. We thus included Δvalue, SumMem and their interaction in regression models accounting for choices and RTs (see supplementary information). We found, again that Δvalue related to choices and RTs when accounting for SumMem. But neither SumMem nor the interaction between Δvalue and SumMem had an effect on choices or RTs.

To further characterize the underlying decision process during the food choices in this experiment, we modeled choices and reaction times using the drift diffusion model (DDM, see supplementary information). We used a hierarchical Bayesian fitting procedure implemented in the HDDM Python package^31^ to estimate regressions for the effects of Δvalue, Δmem, and their interaction on the key DDM free parameters drift rate and start point bias. We find that Δvalue is credibly related to drift rate, as expected. All other effects are non-credible (Fig. S3). This suggests that relative value difference impacts only the speed of evidence accumulation, but relative memorability difference does not impact the decision process.

Together, these results do not support our hypotheses that participants would choose more memorable items, or that participants would make slower choices when options are equally memorable compared to when one item is much more memorable than the other.

##### Relationship between memorability and subjective value

To investigate whether a relationship between subjective value and memorability scores could explain the non-significant results, we explored possible correlations between the two variables across food images. The Pearson correlation between averaged subjective values of food stimuli and the memorability scores showed a non-significant positive correlation (*r*(136) = 0.15, *p* = 0.07), meaning that the memorability scores do not explain much variance in the averaged subjective value of foods in this sample. However, using a larger sample of participants in Lloyd et al.^22^, we found that memorability is correlated with factor loadings on the “tastiness” factor from a factor analysis of all 17 food attribute ratings from the Llyod et al. sample across food items (see supplementary information and Fig. S4).

#### Discussion

The measured memorability of the images of food used in this study was consistent across participants and conformed with the predictions of the DNN model ResMem^19^. However, the memorability scores and the average per-item subjective values were not correlated. These results are in accord with previous literature^9^ that memorability is an independent attribute of visual stimuli that cannot be fully explained by any other attributes.

By design, the difference in values between choice options (|Δvalue|) showed a strong relationship with the choices participants made: participants tended to choose higher-valued items. Based on previous literature that showed people’s value-based choices are highly related to their indicated subjective values of each item^3–5,32,33^, this pattern of results functions as a sanity check for participants’ performance. Moreover, replicating this prior work, we observed a significant inverse relationship between |Δvalue| and RT: individuals tended to respond faster when they encountered choice options that had a larger value difference, and they tended to respond slower when the choice options were close in value.

While our main hypothesis for the study was that individuals would tend to pick more memorable items over less memorable ones if the value difference between items remains relatively close, our results did not show a significant relationship between memorability and choice behavior. There is no evidence to suggest that participants preferred the more memorable items over the less memorable ones while making decisions between food items. In fact, we found substantial evidence in favor of the null hypothesis that there is no effect of memorability on choice. The difference in memorability between choice options is also not related to reaction times while making decisions; two items that are equally memorable are not necessarily harder to choose between. This finding contradicts our prediction that participants would make faster choices if one of the items was much more memorable than the other. Drift diffusion modeling confirmed that memorability did not impact the decision process during value-based choice in this experiment. Additionally, we did not observe a correlation between memorability and the per-item sample-average subjective value placed on food images, suggesting that memorability is dissociable from subjective value. To further our understanding of the effect of memorability on preferences, we next tested whether memorability of semantic representations of food biases peoples’ value-based choices.

## Experiment 2

Since Experiment 1 showed no relationship between image memorability and choice preference for food images, we tested whether the modality of the food items is an important factor in the relationship between memorability and choice. Some of the images used in Experiment 1 may not be representative of the food depicted. Thus, participants may make different decisions when presented with word labels of the choice options where they can picture the food in their mind’s eye compared to when they are presented with potentially unrepresentative images of that food. Therefore, the procedure used for Experiment 2 followed that of the previous experiment except that (1) the stimuli were changed from images to words, and (2) we conducted an additional first experiment (Experiment 2A) with a different participant sample to evaluate how representative a given semantic label was for its corresponding food image. The measure of representativeness ensures that the stimuli used in Experiments 1 and 2 are equivalent and allows us to test whether any effects of value or memorability are related to how well the food images match their corresponding words.

### Experiment 2A: representativeness task

#### Methods

##### Stimuli

We used the same set of food stimuli as in Experiment 1. The words for the food items were coded based on the file name of the images from the original stimulus set^22^. To make the words standardized, we removed the brand names from the word label of the food when they were not necessary for identification (for example, we removed the brand name “Skippy” from peanut butter). All of the words were displayed in lower case, and the length of words ranged from 3 to 34 characters with a mean of 12.5 characters. Some examples of words were pizza, mini muffins, and kit kat (see supplementary information Table S1 for the full list).

##### Participants

Forty participants (48% women, ages ranging between 22 and 39) were recruited on CloudResearch^25^, which is a participant-sourcing platform for online research, and the experiment was conducted through AMT. All participants provided informed consent prior to taking part in the study. All experimental procedures were approved by the University of Chicago IRB and conducted according to approved procedures. The research was performed according to the guidelines and regulations laid out by the IRB.

##### Task

**Figure 4:**
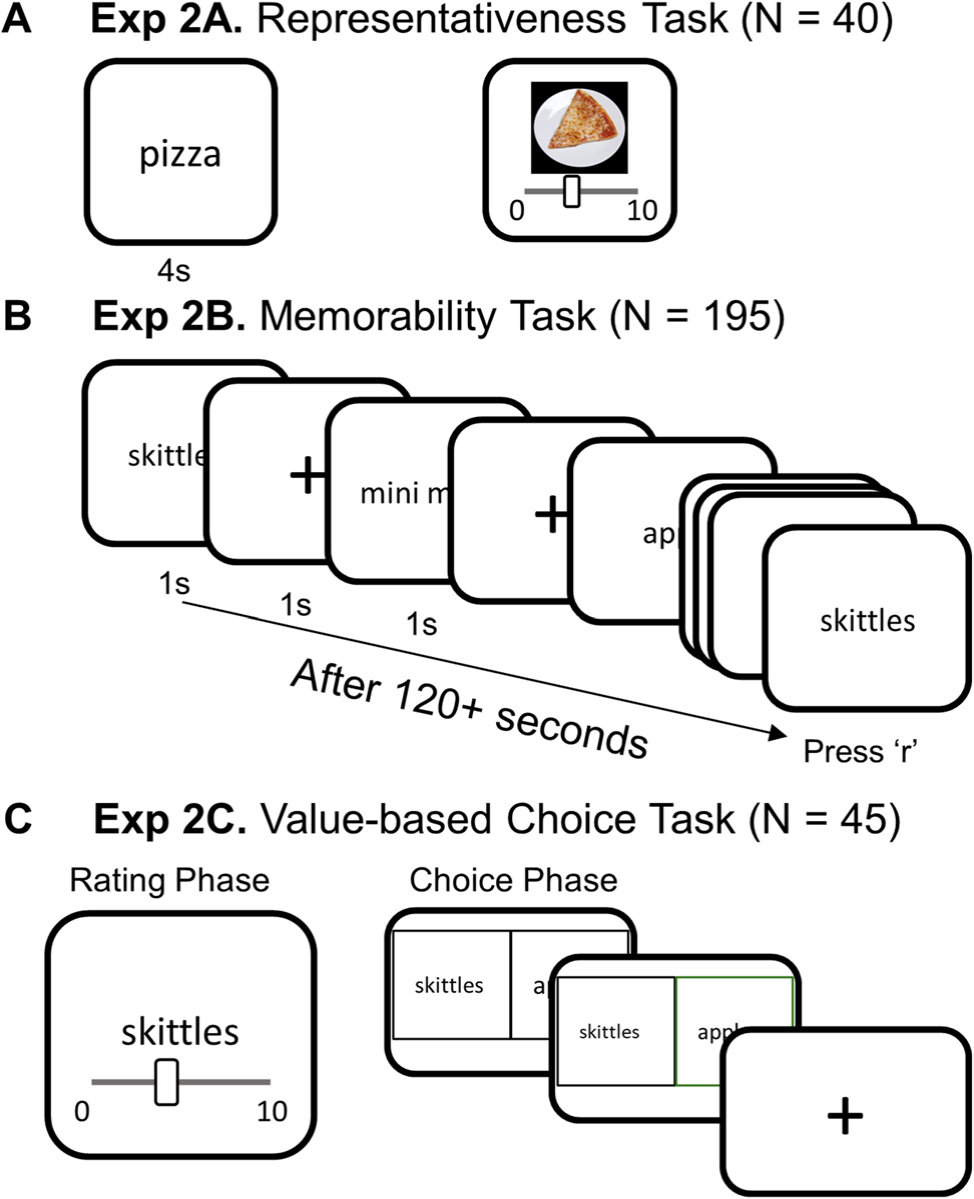
Diagram of tasks in Experiment 2. (**A**) Representativeness task in Experiment 2A. Participants (N = 40) were asked to picture the food depicted by the word in their mind first, then view and rate how representative the presented image was of the food they imagined. (**B**) The memorability task in Experiment 2B was identical to that in Experiment 1A, except the foods were depicted by words, rather than images (N = 195). (**C**) The value-based choice task in Experiment 2C was identical to that in Experiment 1B, except the food choice options were depicted by words, rather than images of food (N = 45). The photograph used in this figure is publicly available for download at https://osf.io/483mx^22^.

To check if the images of food items in Experiment 1 are representative of their word labels used in Experiment 2, we designed a representativeness task and recruited a new sample of participants online. On each trial of the representativeness task, participants saw the name of a food item for 4 s. Participants were asked to picture the food depicted by the word(s) in their mind on a white plate. They were then presented with the image (used in Experiment 1) of the food depicted by the label. Participants were instructed to rate how much the image on the screen matched the food they depicted in their mind. They rated each image on a scale from 0 to 10, with 0 being ‘does not match all’ and 10 being ‘perfect match’ (Fig. 4A).

##### Data analysis

We calculated the z-scored representativeness ratings across items for each participant and averaged the z-scored representativeness rating for each image across all participants.

#### Results

The representativeness scores (*M* = 6.9, *SD* = 1.23) were standardized within each participant and averaged for each image across all participants. The representativeness of images was positively correlated with the average value of images across participants (*r*(136) = 0.18, *p* = 0.03), suggesting that participants tend to evaluate highly representative images as higher valued in general. There was no correlation between the representativeness scores and neither image memorability (*r*(136) = 0.08, *p* = 0.33) nor word memorability (see Experiment 2B, *r*(136) = 0.04, *p* = 0.65), indicating that the memorability of images are not associated with whether the images are representative of the food items at the semantic level.

### Experiment 2B: memorability task

#### Methods

##### Stimuli

The stimuli were the same words as in Experiment 2A.

##### Participants

In Experiment 2B (memorability task), 227 participants were recruited on AMT in order to calculate memorability scores from the average memory response of at least 40 participants for each word stimulus^14^. All participants provided informed consent prior to taking part in the study. All experimental procedures were approved by the University of Chicago IRB and conducted according to approved procedures. The research was performed according to the guidelines and regulations laid out by the IRB. We excluded 32 participants because they performed poorly on the memory task (fewer than 3 hits or 3 correct rejections). This resulted in a final sample of 195 participants (27% women, ages ranging between 18 and 35).

##### Task

The memorability task followed the same procedures in Experiment 1A. The only difference is that each visual image was changed to the word(s) for the corresponding food item (Fig. 4B).

##### Data analysis

We performed the same analysis as in Experiment 1A.

#### Results

**Figure 5:**
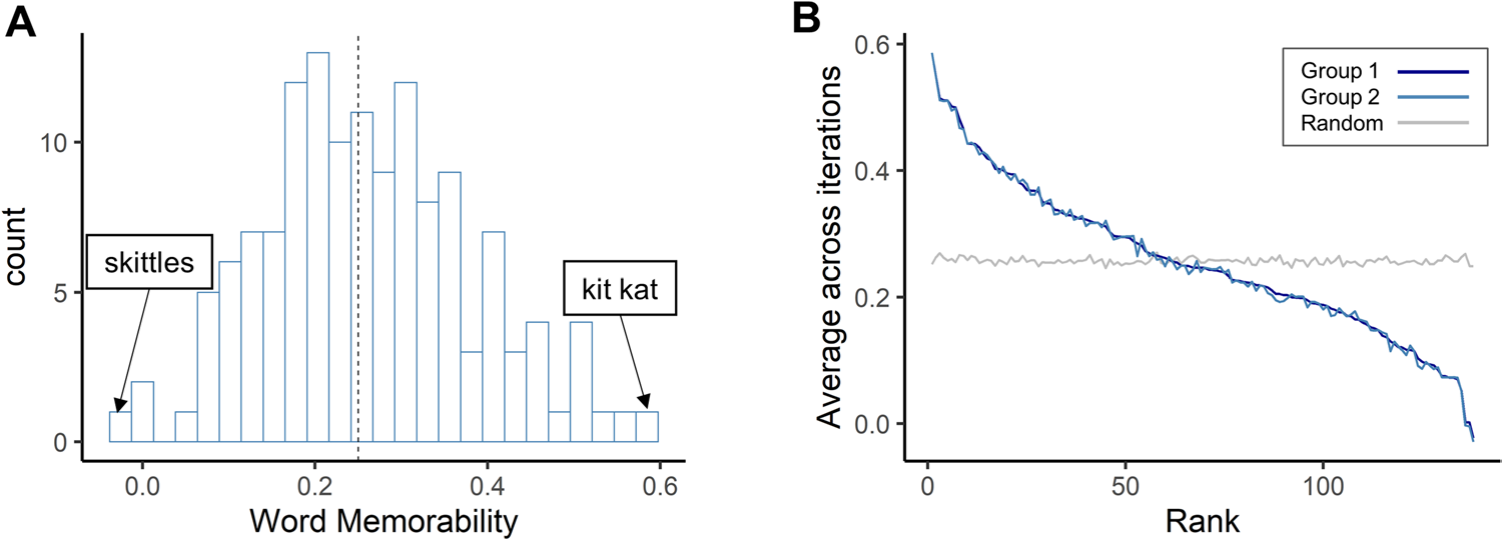
Word memorability scores differ across items but are consistent across participants. (**A**) Distribution of calculated word memorability scores for food items, with examples shown for words with extreme memorability scores. The median of the word memorability scores was 0.25 (dotted vertical line). (**B**) Consistency of word memorability scores in Experiment 2B. The split-half consistency is plotted using ranked average word memorability scores across 1000 iterations of random participant splits. As in Experiment 1A, we plotted the averaged word memorability scores for Group 1 participants (dark blue line) against their rank on the x-axis, scores for Group 2 participants (light blue line), and the scores estimated by random permuted chance (grey line) against the same memorability rank. Group 1 and Group 2 show significant consistency in which words participants remembered and forgot.

The calculated word memorability scores varied across items (*M* = 0.26, *SD* = 0.12, Fig. 5A). The minimum score was -0.03 for “skittles”, and the maximum score was 0.59 for “kit kat”. The split-half consistency analysis indicated that the word memorability scores were consistent across participants (*ρ* = 0.25, *p* = 0.01, Fig. 5B); people remembered and forgot the same food words.

### Experiment 2C: value-based choice task

#### Methods

##### Stimuli

The stimuli were the same as in Experiments 2A and 2B.

##### Participants

In Experiment 2C (value-based choice task), 59 participants were recruited. All participants provided informed consent prior to taking part in the study. All experimental procedures were approved by the University of Chicago IRB and conducted according to approved procedures. The research was performed according to the guidelines and regulations laid out by the IRB.

Fourteen participants were excluded based on the same exclusion criteria as in Experiment 1: three participants were excluded because their probability of choosing items on the right was 2 standard deviations away from the mean across participants, indicating that they were not choosing based on their personal preferences, but instead pressing the same button on every trial. As in Experiment 1B, we calculated delta value (Δvalue_word_), and performed logistic regressions to test the effect of Δvalue_word_ on choice preferences for each participant separately. Eleven participants were excluded because they had negative parameter estimates or a non-significant effect of Δvalue_word_ on choices, which indicates that their choice preferences did not conform to the subjective preferences they stated during the rating phase. This resulted in a final sample of 45 participants (45% women, ages ranging between 22 and 48).

##### Task

The value-based choice task followed the same procedures as in Experiment 1B. The only difference was that each visual image was changed to the word(s) for the corresponding food item (Fig. 4C).

##### Data analysis

We performed the same analyses as in Experiment 1B.

#### Results

##### Relationship between value and choice

**Figure 6:**
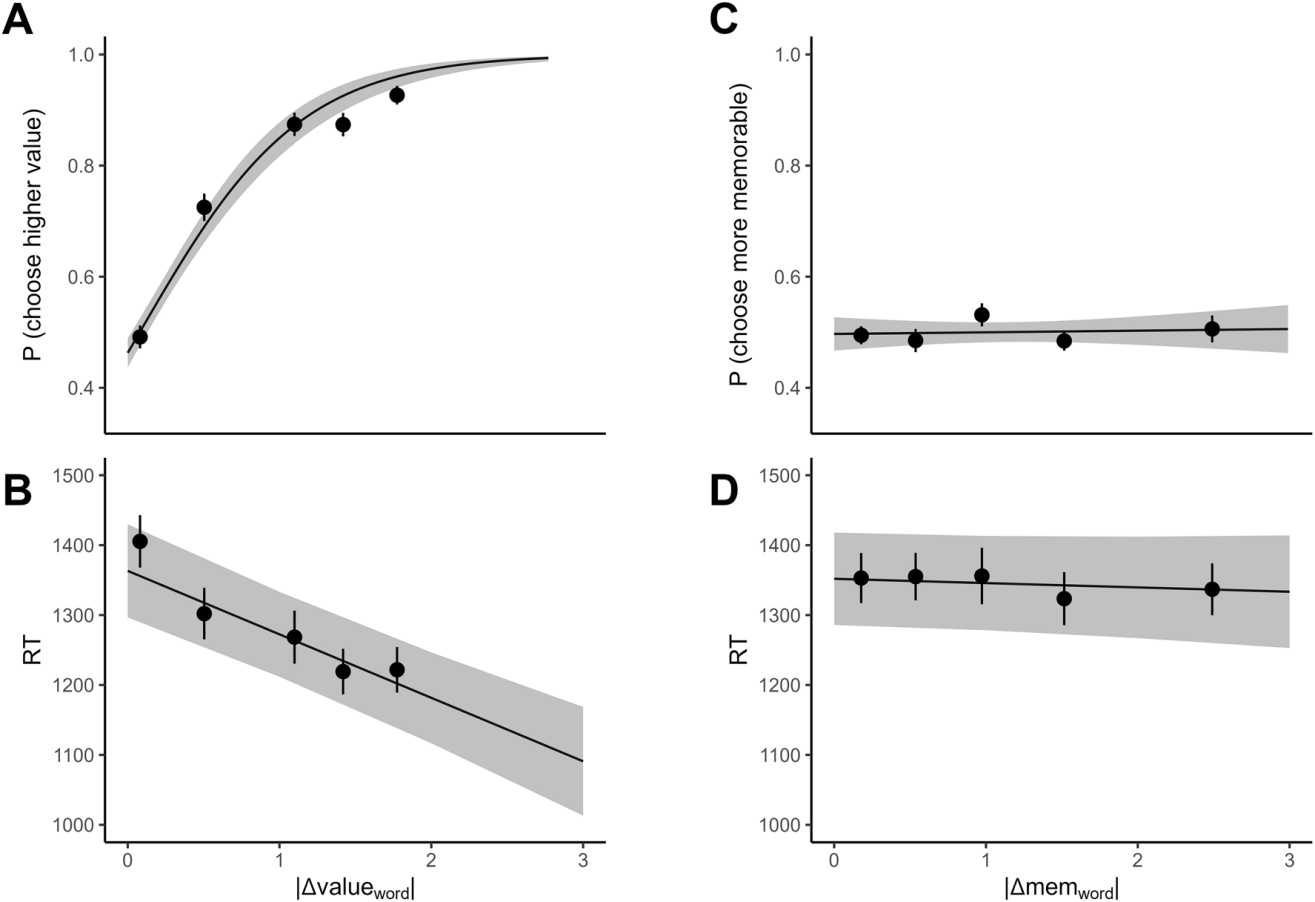
Subjective value, but not word memorability, relates to choice behavior and speed. The proportion of more-valuable item choice (**A**) and RT (**B**) plotted as a function of |Δvalue_word_| for trials where |Δmem_word_| was low or near zero. The proportion of more-memorable item choice (**C**) and RT (**D**) plotted as a function of |Δmem_word_| for trials where |Δvalue_word_| was low or near zero. All of the models had 3080 trials. In each plot, the solid black line represents the predicted regression line. The shaded gray area represents the 95% confidence intervals. The trial data were binned into 5 equal-sized bins on the x-axis. The black dots represent the mean proportion of choosing a higher-valued item (top-left), choosing the more memorable item (top-right), or mean RT (bottom) across participants within bins. Error bars are standard error of the mean across participants within bins.

Like in Experiment 1, we first tested the relationship between choices and item value. To control for the possible effect of memorability on choices, we selected trials where |Δmem_word_| was low or close to zero using a median split. The mixed-effects logistic regression showed that value was related to choice behavior (*OR* = 6.56, 95% *CI* = 5.05–8.51, *p* < 0.001), meaning that the value placed on food words predicts food choices (Fig. 6A). Note that this relationship is necessarily significant because of our exclusion criteria. In the linear mixed-effects model of RT, |Δvalue_word_| was significantly negatively related to RT (*β* = -0.06, 95% *CI* = − 0.08 to − 0.05, *p* < 0.001, Fig. 6B), which is consistent with previous literature and Experiment 1B. This finding confirms that individuals tend to spend more time deliberating when faced with choice options that are close in value^4,5,32,34,35^. The effect of Δvalue_word_ on choices and RT is significant when accounting for Δmem_word_ when both are included in the same model to account for all trials (see supplementary information). The significant relationship between |Δvalue_word_| and choices (as well as RT) replicate well-known effects and indicate that participants were actively engaged in the word choice task.

##### Relationship between memorability and choice

We tested the effect of |Δmem_word_| on participants’ choices and RTs to test whether word memorability influences item choice. We controlled for the influence of value difference on choices by selecting trials where |Δvalue_word_| was close or equal to zero. Consistent with what we found in Experiment 1B, |Δmem_word_| was not related to choice behavior (*OR* = 1.01, 95% *CI* = 0.93–1.10, *p* = 0.79, Fig. 6C). Using a Bayesian model fitting procedure, we replicated the same effect size and calculated the Bayes Factor for the model. For this model, BF01 = $2.91\times {10}^{4}$, providing overwhelming evidence in favor of the null hypothesis that |Δmem_word_| had no effect on choice (see supplementary information). In addition, |Δmem_word_| was not related to RT (*β* = -0.004, 95% *CI* = − 0.01 to 0.01, *p* = 0.46, Fig. 6D). The calculated Bayes Factor for the regression model testing the effect of |Δmem_word_| on RT was BF01 = $9.71\times {10}^{5}$, again providing very strong evidence in favor of the null hypothesis that |Δmem_word_| is not related to RT (see supplementary information). These results replicate the non-significant findings in Experiment 1B and do not support our hypothesis that participants may prefer more memorable food items and make choices slower if the memorability scores for food items are close to one another.

##### Relationship between memorability and subjective value

As in Experiment 1B, we explored possible correlations between the subjective value placed on the word description of foods and word memorability across food items. The Pearson correlation showed a non-significant correlation (*r*(136) = 0.02*, p* = 0.83), meaning that the memorability scores for words could not explain variance in the averaged subjective value of words in this sample.

#### Discussion

The words used in Experiment 2 to describe the images of food used in Experiment 1 were largely representative of the latter. More representative word-image pairs were rated higher on value by participants, but representativeness was not associated with either word or image memorability. These results further support prior findings that memorability is an attribute that differs from semantic information or subjective preferences^9^.

Compared to image memorability (Fig. 2A), word memorability (Fig. 5A) scores were lower on average, but still showed high variance, with some words having high memorability scores, and others having very low memorability scores. Furthermore, like for the images, memorability scores for the words were highly consistent (Fig. 5B). This suggests that the memorability of semantic information—like image memorability—is also a stimulus-based attribute that is independent from personal experiences.

The results of Experiment 2B and 2C fully replicated those of Experiment 1. The difference in values between the choice options showed expected and characteristic relationships with choices and RT. We had predicted that participants would tend to choose more memorable words than forgettable ones when the values of the two options were similar. But no evidence was found to support this prediction. These results support an account that memorability has no influence over value-based decisions.

## General discussion

The purpose of the current work was to quantify the influence of item memorability on value-based choices. We hypothesized that more memorable items are embedded within a richer associative network, and when presented as a choice option, individuals may draw more value-relevant associations with that item to guide choice. Thus, we predicted that individuals would prefer more memorable over less memorable items when the subjective values placed on the choice options are similar. To test the hypothesis, we measured the memorability scores for food stimuli and conducted a choice task in which participants indicated their preferences amongst pairs of items that differed little or significantly in memorability scores and subjective values. Surprisingly, replicating across different stimulus formats (images and words), we did not find a relationship between item memorability and the value-based choices participants made. Although participants were highly consistent in what food items they remembered and forgot, their choice behavior did not reflect these stimulus-based memory effects.

One possibility is that memorable items may not be positively valenced, and thus not preferred, even though they are prioritized in memory. For example, more negative attributes are correlated with face memorability while positive attributes are not^12^. It could be that items on the edge of a conceptual map that choice options can be placed on (e.g., in semantic space) are more memorable regardless of valence and therefore would not drive choices uniformly. Furthermore, one study has shown that imbuing a stimulus with reward value does not alter its memorability^13^. Even when participants are rewarded with money to remember and forget certain images, the original memorability of the image has a stronger influence on individuals’ memory of the image than the reward ascribed to it. These results suggest that the personal memories of value elicited by the stimuli in the experiment might not interact with the memorability of stimuli to influence memory processes. Findings in the current study of no relationship between memorability and value-based choice as well as no correlation between average subjective value placed on items and item memorability add evidence to these previous accounts that stimulus memorability is dissociable from valence, subjective value, or preference.

Another potential reason why memorability does not influence value-based choice is the stimulus-intrinsic nature of memorability. If memorability is determined purely by perceptual features of the stimulus, then we should not expect it to influence value-based decisions. Although memorability has been linked to perceptual features of stimuli, work focused on uncovering the determinants of image memorability has shown that the latter is not well accounted for by low-level visual features such as color, contrast, or edges^15^. Furthermore, memorability is more predictable by a combination of stimulus features including more abstract semantic features that can be extracted from images such as high-level category or function^36^. Similarly, a neural network model (ResMem) that includes deeper, more semantic, processing has shown the best success at predicting memorability when compared to shallower networks^19^. Thus, given that memorability is not purely a perceptual feature, we reasoned that it would influence value-based decisions even if some of the features that determine memorability are shared with those that determine subjective value. Indeed, the average subjective value placed on items is not correlated with item memorability in both experiments, suggesting that there is little overlap in the constellation of features that govern memorability and subjective value. However, our findings suggest that the unique determinants of memorability do not influence value-based choice behavior.

Although we do not observe a relationship between stimulus memorability and choice behavior here, extensive prior work has shown a link between memory and decision making^5,8,20,21,37–44^. In particular, recent work has characterized a memory bias on value-based choice when the choice options must be recalled from memory^40–43^. In the current study, participants were neither instructed nor required to rely on memory to make food choices. Other work has identified an important role for memory in open-ended decisions (e.g., where to eat) where the choice set is not imposed by the experimenter, but rather self-generated (e.g., a list of fast-food chains)^44^. However, in that study, the memorability of the logos of the fast-food restaurants participants included in their self-generated choice set, for example, was not examined. Given this rich literature that links memory to value-based decisions and the current work that shows no influence of item memorability on value-based choice, there may be two dissociable aspects of memory: the specific memory experiences of an individual, and the intrinsic influence on memory of the stimulus (i.e., memorability). The effect of memory on decision making could be driven by individuals’ specific memories, such as their prior experiences with those food items, or the contexts in which they learned about those food items. In contrast, global stimulus memorability (which stimuli are easier to remember or forget) may have less influence on individuals’ decisions. Indeed, key differences have been observed when comparing participant-based memory and stimulus-based memorability in other work. For example, for scene and face images, there are low or no correlations between true image memorability and what people think they will remember^15,23^, indicating that memorability is dissociable from individualized inferences about memorability. Other work has shown that an individual’s current attentional state and image memorability are independent and non-interacting predictors of memory^45^. Furthermore, neuroimaging work has identified separable patterns in the brain for stimulus memorability and markers of memory in a given individual^16,23^. These results, in combination with the current work, support the idea that memory retrieval processes involved in making decisions might be distinct from retrieval of the features that make certain stimuli memorable or forgettable. Finally, it is thought that the construction of subjective value may rely on personal memory^6,37,40,46,47^, rather than on stimulus memorability. Therefore, memorability may be a global stimulus feature that helps to organize and prioritize perceptual information in memory, but decision making is driven by more idiosyncratic or experience-based memories at the individual level.

The current study presents some limitations that can guide future research. First, the study was conducted online, and participants made hypothetical decisions that were not incentive compatible; participants were not offered a food chosen during the task as a snack to eat at the end of the experiment. In contrast, in the value-based choice task conducted by Bakkour et al.^5^ and others, participants had a chance to receive the actual food item as a snack based on their indicated subjective values after the rating, which may have motivated participants to make more veridical evaluations about their preference of each food item. Future in-person studies that better incentivize participants and encourage them to pay full attention to the task are needed to solidify conclusions from the null effect of memorability on value-based choices found in the current study. Second, we used food stimuli in this study because people have strong pre-existing subjective preferences for food. However, the subjective values placed on food might be too strong a determinant of choice that they override a moderate, if any, effect of memorability on choice. Future studies are needed to investigate the effect of memorability on choice in a reward learning design: participants might have better performance in associating rewards with memorable items versus forgettable ones, and make subsequent decisions differently. Future studies should also replicate these effects across other stimulus types beyond food images and names, to avoid potential behaviors or processes that are specific to food-related decisions.

Despite these limitations, the current research can be seen as a first step towards integrating two lines of research—value-based decision making research and the field of memorability. To our knowledge, the effect of memorability on value-based choice has not been previously investigated. The finding that value-based choices and reaction times are largely driven by subjective value of foods is consistent with previous literature^3–5,32,48^, suggesting that people spend more time deliberating their choice when the values of choice options are similar. Also consistent with previous work, the measure of food item memorability was highly consistent across participants using both image and word stimuli. Measured image memorability was consistent with predictions from a neural network, demonstrating that memorability is a robust attribute of these stimuli^12,14,19^. However, the crucial test of the hypothesized effect of memorability on value-based choice did not conform to our predictions.

Finding no relationship between memorability and choice is informative and useful. Beyond the contribution to understanding the role of memory in decision making processes, these results have important implications for the real world. The results of this study suggest that people’s value-based decisions are not biased by more memorable environments (or advertisements)—individuals do not tend to choose more memorable options when asked about their preferences. Also, our results show that memorability is unlikely to be a confound in value-based choice tasks. While the specific images and words that we use may have a strong influence on our memories, those same forces do not appear to influence our decisions.

## Supplementary Information

Supplementary Information.
