# Supplementary material for: Item memorability has no influence on value-based decisions
Source: Sci Rep. 2022 Dec 21;12:22056. doi: 10.1038/s41598-022-26333-5 (PMC9772201; doi:10.1038/s41598-022-26333-5)
Supplement: Supplementary file 1 — Supplementary Information. [file 41598_2022_26333_MOESM1_ESM.docx]

**Item memorability has no influence on value-based decisions**

Xinyue Li^1^, Wilma A. Bainbridge^1,2^, Akram Bakkour^1,2^

^1^Department of Psychology, University of Chicago

^2­­^Institute for Neuroscience, University of Chicago

**Supplementary information**

***Expanded regression models.*** Instead of splitting the trials based on the value of Δvalue or Δmem, we included all of the trials in Experiment 1B in the same model. To investigate the possible interaction between Δvalue and Δmem in predicting choice behavior, we entered Δvalue and Δmem as fixed effects and interaction between the two variables in the mixed-effects logistic regression model. For the random effects, we included the intercept by subjects, and by-subject random slopes for Δvalue, Δmem, and the interaction.

The result showed that Δvalue was related to the probability of choosing items on the right-hand side (*OR* = 3.32, *CI* = 2.81 – 3.92, *p* < .001), and Δmem was not related to choice behavior (*OR* = 1.00, *CI* = 0.96 – 1.04, *p* = 0.91). We also did not find an interaction effect between Δvalue and Δmem (*OR* = 1.00, *CI* = 0.94 – 1.05, *p* = 0.94). These results suggested that the value difference between the two items significantly predicted participants’ choice of items, whereas neither the memorability difference between items nor the interaction between memorability and value differences could predict choice behavior.

A linear mixed-effects model was used to test if the interaction between Δvalue and Δmem is related to RT using all of the trials in Experiment 1B. To predict the log-transformed RT in the linear mixed-effects model, we entered |Δvalue| and |Δmem| as fixed effects, and the interaction between the two variables. For the random effects, we entered intercept by subjects, and by-subject random slopes for |Δvalue|, |Δmem|, and the interaction.

There was a significant relationship between RT and |Δvalue| (*β* = -0.04, *CI* = -0.06 – -0.02, *p* < .001). But no relationship was found between RT and |Δmem| (*β* = -0.001, *CI* = -0.02 – 0.01, *p* = 0.27) nor the interaction of |Δvalue| and |Δmem| (*β* = 0.001, *CI* = -0.01 – 0.01, *p* = 0.86). The results from the linear mixed-effects model suggested that an increase in |Δvalue| can predict a decrease in response time. Contrary to our prediction, the results do not support the hypothesis that participants tend to respond faster when |Δmem| is larger, and there is no interaction between |Δvalue| and |Δmem| in predicting RT.

We also ran the same analysis with a sample of 61 participants that included participants who were excluded based on predefined exclusion criteria, but excluded those who failed the attention checks. Trials that have RT lower than 300 ms were excluded from the analysis because participants could not have made value judgments on those trials in such a short time. Similar to Exp1B, we calculated |Δvalue| and |Δmem| for each trial, and fitted regression models to test the relationship of the two variables on choice preference and RT. All of the models had 3856 trials. The results did not change qualitatively when participants were not excluded. Among trials that had low or close to 0 |Δmem|, |Δvalue| was significantly related to the choice preference (*OR* = 2.62, *CI* = 2.12 – 3.23, *p* < .001), meaning that participants tend to choose higher value items over the ones with lower values. |Δvalue| can also predict the RT **(***β* = -0.03, *CI* = -0.04 – -0.01, *p* < .001), where participants tend to make choices more slowly when the difference of values between the items are close compared to when one of the items is much more valuable than the other. For trials that low or close to 0 |Δvalue|, there was no relationship between |Δmem| and choice preference (*OR* = 1.00, *CI* = 0.93 – 1.07, *p* = 0.93), or RT (*β* = -0.01, *CI* = -0.02 – 0.002, *p* = 0.09).

***Power*** ***analysis for the effect of memorability on choice.*** A post hoc power analysis was conducted using simr package[^1^](https://app.readcube.com/library/0f0e6e3f-9bb4-47c8-b2a5-c2f0a71803b0/all?uuid=26652458471026275&item_ids=0f0e6e3f-9bb4-47c8-b2a5-c2f0a71803b0:7211e08c-1409-42d8-9211-de2aac5e2ad8) to estimate the power of the mixed-effects logistic regression model. Both Δmem and Δvalue were applied as the fixed effects to predict the probability of choosing the food item on the right-hand side, with random effects of intercept and slope by subject. The power estimation of Δmem used an alpha of .05. Simulated power for a small effect size (OR=1.68)[^2^](https://app.readcube.com/library/0f0e6e3f-9bb4-47c8-b2a5-c2f0a71803b0/all?uuid=2536854878417466&item_ids=0f0e6e3f-9bb4-47c8-b2a5-c2f0a71803b0:fc4b986f-3ca9-4284-b171-2df93d5842c2) with N=44 participants was above 99%, suggesting we were well-powered to detect a small effect of Δmem on choice with the current sample size. In fact, the smallest detectable effect size with this sample at a power of 80% was OR=1.067. We also simulated power to detect the measured effect size in this experiment across a range of simulated sample sizes and found that power ranged from 4% to 5.5% with a sample as large as 500 participants (Figure S1).


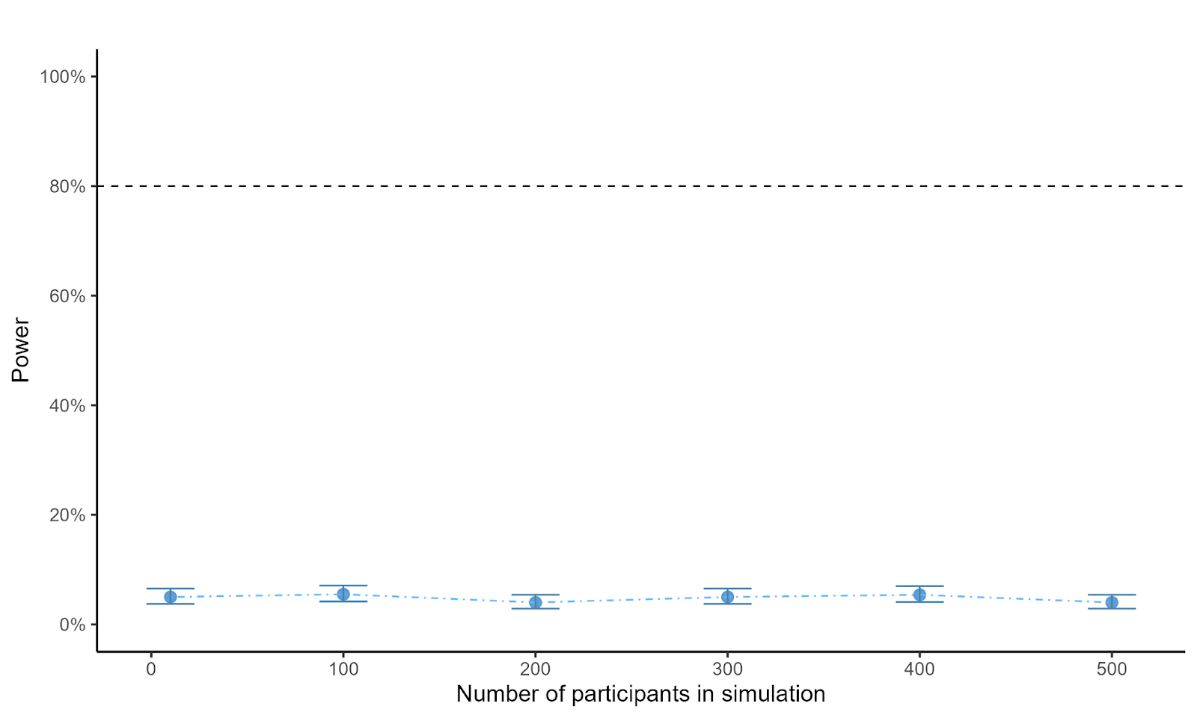


***Figure S1*.** Power curve for Δmem in the mixed-effects logistic regression model that included Δmem and Δvalue predicting the probability of choosing items on the right. Error bars represent 95% confidence intervals for estimated power. The dashed black line represents a power of 80%.

***Bayesian model fitting.*** To determine the usefulness of the null results from the mixed-effects logistic regression model, a Bayesian generalized regression model was applied using the brms package[^3,4^](https://app.readcube.com/library/0f0e6e3f-9bb4-47c8-b2a5-c2f0a71803b0/all?uuid=45004181130245935&item_ids=0f0e6e3f-9bb4-47c8-b2a5-c2f0a71803b0:19f9ebb2-b590-45bc-863a-48f078c6b72b,0f0e6e3f-9bb4-47c8-b2a5-c2f0a71803b0:15c4991f-f90e-4635-836b-5d071081521b). The prior distributions for the intercept and the fixed effects of |Δmem| were normal distributions centered at 0, with a standard deviation of 10. The prior distribution for the standard deviation was a Cauchy distribution centered at 0 with a scale of 10. The results showed that the estimate for the fixed effect of |Δmem| on choice was Odds Ratio (OR)=0.99 (*Credible Intervals* = 0.92– 1.07). We compared the fit of the data under the null hypothesis and the alternative hypothesis. An estimated Bayes factor that provides evidence in favor of the null (i.e., BF01) was $3.70\times{10}^{4}$ times more likely under the null hypothesis than under the alternative hypothesis. To ensure that our choice for the width of prior distributions did not lead our inference astray, we refit the model using different prior widths and found that, even for narrow prior distributions BF01 provides strong evidence in favor of the null (Figure S2). Reported BF01 values in the main text were calculated based on models for which the prior distributions had a specified standard deviation of 10.

Similarly, we applied Bayesian modeling to test the effect of memorability on RT. The results showed that the estimate for the fixed effect of |Δmem| was close to 0 (*Credible Intervals* = -0.02 – 0.01). We calculated the Bayes Factor to compare the fit of the data under the null hypothesis and the alternative hypothesis that |Δmem| and RT are related. For the RT model BF01 = $1.40\times{10}^{6}$, suggesting strong evidence for the null hypothesis.


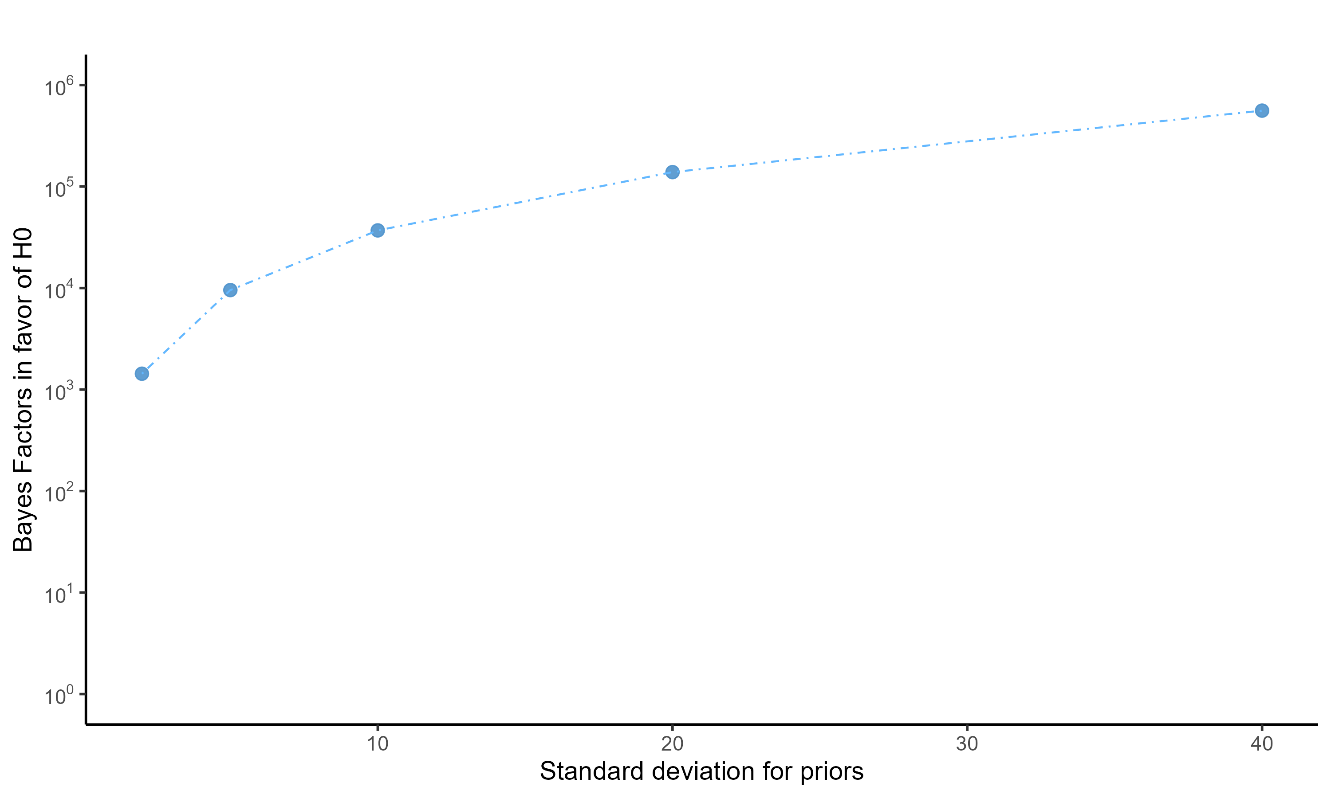


***Figure S2*.** Bayes Factors in favor of the null (BF01) for the regression model testing the effect of |Δmem| on choice preference as a function of the standard deviation of prior distributions. Tested standard deviations were between 2 and 40. BF01 values are plotted on a log scale.

***Drift diffusion modeling.*** We simultaneously modeled choice and reaction time (RT) data in Experiment 1B using the drift-diffusion model (DDM). The DDM assumes evidence (in this case value- and/or memorability-relevant evidence) is sequentially sampled, accumulated and integrated over time until a threshold is reached to make a decision. We modeled the data with four free parameters: the start point bias (*z*) governs at what level evidence accumulation starts (i.e., is one decision favored over another at the start of evidence accumulation), the drift rate (*v*) governs the rate of evidence accumulation, the bound height (*a*) governs the amount of evidence needed to commit to a decision, and non-decision time (*t*) is the modeled portion of reaction time that does not contribute to the decision process (e.g., time for perceptual processing of the stimulus and motor execution). Model parameters were estimated using hierarchical Bayesian estimation as implemented in the HDDM python package[^5^](https://app.readcube.com/library/0f0e6e3f-9bb4-47c8-b2a5-c2f0a71803b0/all?uuid=7417651180596297&item_ids=0f0e6e3f-9bb4-47c8-b2a5-c2f0a71803b0:6dc34c37-c7fe-43e2-97e0-3ff6eef7ba45). Markov Monte Carlo sampling with 20,000 samples and 2,000 burn-in was used to estimate the posterior distributions of all parameters. We modeled *z* and *v* as a function of |Δvalue|, |Δmem|, and the interaction between the two using the HDDMRegressor function.

As expected, we found that |Δvalue| credibly impacted drift rate *v* (mean regression coefficient = 0.75, credible interval = 0.65 – 0.84, Figure S3 left), but did not impact start point bias *z* (mean regression coefficient = -0.01, credible interval = -0.033 – 0.008, Figure S3 right). Furthermore, |Δmem| had no impact on either *v* (mean coefficient = 0.008, credible interval = -0.07 – 0.08, Figure S3 left) or *z* (mean coefficient = 0.009, credible interval = -0.009 – 0.016, Figure S3 right). There were no credible effects of the interaction between |Δvalue| and |Δmem| on drift rate or start point bias.

We conclude from this analysis that memorability neither biases the accumulation of evidence a priori (which would be reflected in a relationship between |Δmem| and start point bias *z* estimated by DDM) nor does it influence the quality of evidence accumulation (which would be reflected in the relationship between |Δmem| and drift rate *v* estimated by DDM). These results suggest that memorability in this study does not play a role in the decision process when individuals are choosing what to eat.

**
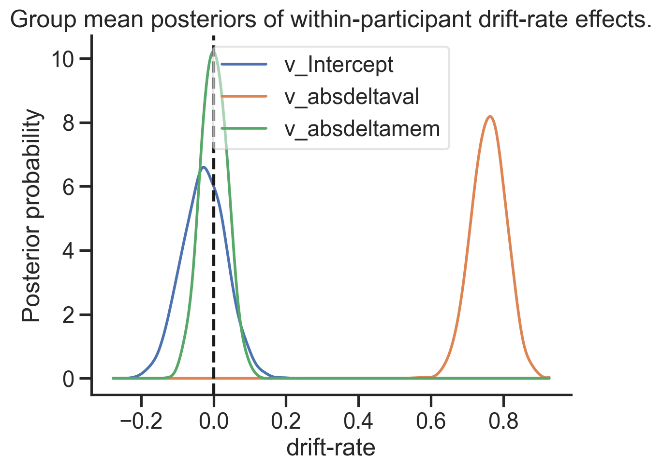

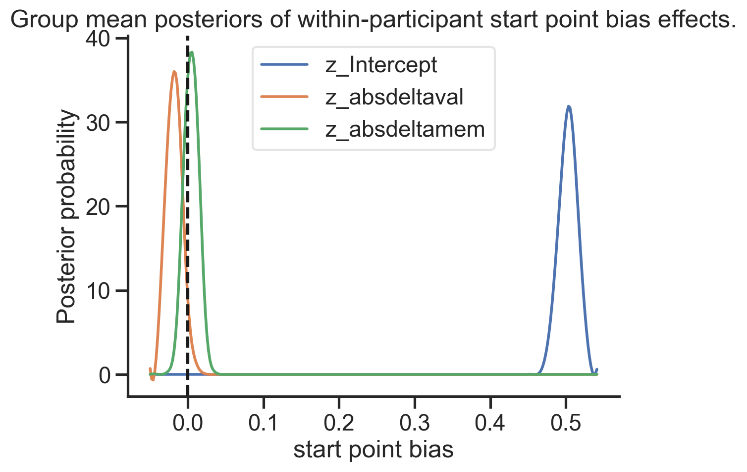
**

***Figure S3*.** Posterior distribution of the effect of the intercept (blue), |Δvalue| (orange) and |Δmem| on drift rate (*v*, left) and start point bias (*z*, right). The dashed line indicated 0 (no effect of the regressor on the fitted free parameter). The entire distribution for the effect of |Δvalue| on drift rate (*v*) is to the right of zero, indicating very strong evidence for a relationship between |Δvalue| and drift rate. No other credible effects of |Δvalue| or |Δmem| were observed.

***Effect of sum of memorability on choice behavior.*** A mixed-effects logistic regression model was applied to explore if the sum of memorability scores for the two choice options (SumMem) predicts choice behavior with all of the trials in Experiment 1B. We entered SumMem and Δvalue as fixed effects to predict the probability of choosing items on the right-hand side. We also entered an interaction effect between the two variables, and for random effects, we included intercept by subjects and by-subject random slopes.

The results showed that Δvalue can predict choice preferences (*OR* = 2.90, *CI* = 2.69 – 3.13, *p* < .001), meaning that participants tend to choose items with higher values. However, there was no relationship between the SumMem and choices (*OR* = 1.01, *CI* = 0.96 – 1.06, *p* = 0.76), and there was no interaction between Δvalue and SumMem in predicting choice preference (*OR* = 0.97, *CI* = 0.50 – 1.19, *p* = 0.23).

It is possible that when SumMem is low, participants may have a harder time retrieving information that could be entered into a decision process, which would lead to longer RTs on those trials. To test this hypothesis, we applied a mixed-effects regression model that included SumMem and |Δvalue| as fixed effects, and we entered an interaction of the two variables to predict the log-transformed RT for all of the trials in Experiment 1B. The result showed that |Δvalue| was significantly related to RT (*β* = -0.04, *CI* = -0.06 – -0.02, *p* < .001), meaning that participants made faster decisions when choosing between items that have greater value differences. But we did not find a significant relationship between SumMem and RT (*β* = -0.01, *CI* = -0.01 – 0.001, *p* = 0.08). There was no interaction of |Δvalue| and SumMem on RT (*β* = 0.0004, *CI* = -0.01 – 0.01, *p* = 0.90). These results suggest that SumMem was not related to choice behavior, and the difference in value between choice options was the main factor that relates to choice preferences and RT.

***The relationship between ResMem and choice behavior*.** To investigate if the memorability scores predicted by ResMem were related to the choice of food items, we applied ResMem to get predicted memorability scores for all of the 138 food images[^6^](https://app.readcube.com/library/0f0e6e3f-9bb4-47c8-b2a5-c2f0a71803b0/all?uuid=9471441291399483&item_ids=0f0e6e3f-9bb4-47c8-b2a5-c2f0a71803b0:41915360-7912-46f3-a62b-dc2785bc841e). For each trial in Experiment 1B, we calculated ΔResmem, which is the z-scored ResMem score for the food image on the right side minus that on the left side of the screen. We also calculated the absolute value of ΔResmem (|ΔResmem|).

A mixed-effects logistic regression model was applied to test the effect of ΔResmem and its interaction with Δvalue on the probability of choosing the item on the right side using all of the trials in Experiment 1B. We entered fixed effects of ΔResmem and Δvalue and an interaction of the two variables. For the random effects, we entered intercept by subjects and by-subject random slopes. The multiple regression model showed that participants tend to choose higher-valued items (*OR* = 3.33, *CI* = 2.82 – 3.94, *p* < .001). We also found a significant negative relationship between ΔResmem and choice preference (*OR* = 0.94, *CI* = 0.89 – 0.99, *p* = 0.02)， meaning that participants tend to choose food images that had a lower memorability score predicted by Resmem when accounting for Δvalue. But no interaction effect was found for ΔResmem and Δvalue (*OR* = 0.99, *CI* = 0.96 – 1.02, *p* = 0.34).

A mixed-effects multiple regression model was applied to test if the predicted memorability from Resmem is related to participants’ RT. More specifically, we put the |ΔResmem| and |Δvalue| as the fixed effects and an interaction of the two variables to predict the log-transformed RT in the linear mixed-effects model. We also entered random intercepts for subjects and by-subject random slopes for the two variables and the intercept. The result showed a significant relationship of |Δvalue| on RT (*β* = -0.05, *CI* = -0.07 – -0.03, *p* < .001), suggesting that participants were faster when facing choices between items that differed significantly on value. However, the absolute difference of predicted memorability from Resmem was not related to participants’ RT (*β* = -0.005, *CI* = -0.002 – 0.01, *p* = 0.44). We also did not find a significant effect of the interaction between |ΔResmem| and |Δvalue| on RT (*β* = 0.009, *CI* = -0.001 – 0.02, *p* = 0.08).

Largely consistent with results using measured item memorability, these results showed that participants tend to choose items with a lower predicted memorability scores from Resmem, but their RT was not influenced by the Resmem scores.

***Relationship between memorability and other food attributes.*** The images used in the current study are part of a stimulus set published by Lloyd and colleagues^7^. In the original study by Lloyd et al.[^7^](https://app.readcube.com/library/0f0e6e3f-9bb4-47c8-b2a5-c2f0a71803b0/all?uuid=3672336656946944&item_ids=0f0e6e3f-9bb4-47c8-b2a5-c2f0a71803b0:afc19b81-b343-4c4a-a1a6-b2c9397951c7), each image was rated on 17 different subjective attributes (e.g., tastiness, healthiness, etc.) by a large U.S. sample on Amazon Mechanical Turk (AMT, *N* = 1054). Data provided with the stimulus set also included objective measures of perceptual features of the images (e.g., brightness, contrast, etc.) and nutritional information of the foods depicted in the images (e.g., calories, fat, etc.) estimated by professional nutritionists.

To better understand the relationship between memorability scores and other attributes of the food items, we performed exploratory analysis relating memorability to the subjective ratings and objective values obtained from the Food Folio dataset[^7^](https://app.readcube.com/library/0f0e6e3f-9bb4-47c8-b2a5-c2f0a71803b0/all?uuid=3672336656946944&item_ids=0f0e6e3f-9bb4-47c8-b2a5-c2f0a71803b0:afc19b81-b343-4c4a-a1a6-b2c9397951c7). The ratings in the Food Folio dataset were filtered to only include data from participants aged between 18-35 to match the age range of Experiment 1.

Exploratory factor analysis (EFA) was also performed to determine the main factors that group the food attributes together using the averaged values across participants for each image. Following the EFA conducted by Lloyd and colleagues, this study also used the varimax rotation method for the filtered dataset so that the cross-factor loadings can be minimized. The number of factors was determined based on the Cattell-Nelson-Gorsuch (CNG) scree test[^8^](https://app.readcube.com/library/0f0e6e3f-9bb4-47c8-b2a5-c2f0a71803b0/all?uuid=9306080021905149&item_ids=0f0e6e3f-9bb4-47c8-b2a5-c2f0a71803b0:f7838e19-1cd0-4691-bed6-2fff44134ba6), which was conducted using the nCng function in the nFactors package[^9^](https://app.readcube.com/library/0f0e6e3f-9bb4-47c8-b2a5-c2f0a71803b0/all?uuid=8632242315381294&item_ids=0f0e6e3f-9bb4-47c8-b2a5-c2f0a71803b0:78259202-c926-4b86-9c25-c8bad8a277a2). The underlying factors of the 17 attribute ratings were identified by loadings of the attribute ratings on the factors. To consider an attribute to load on a given factor, 0.5 was set as the threshold. These factor loadings were used in Experiment 1 to examine correlations between memorability and subjective evaluations of these foods. Exploratory factor analysis in the Food Folio dataset identified three factors that mainly explain the variances: healthiness, tastiness, and umami taste[^7^](https://app.readcube.com/library/0f0e6e3f-9bb4-47c8-b2a5-c2f0a71803b0/all?uuid=765882326030324&item_ids=0f0e6e3f-9bb4-47c8-b2a5-c2f0a71803b0:afc19b81-b343-4c4a-a1a6-b2c9397951c7).

To further explore which attribute of the food items is most related to the memorability of the food images, we performed multiple regression analyses on memorability with the main factors from the Food Folio dataset as variables of interest. The results of the regression indicated that only tastiness significantly correlated with memorability scores (*β* = 0.083, *p* = 0.004; Figure S4), but there was no relationship between memorability scores and healthiness (*β* = -0.009, *p* = 0.54) nor umami taste (*β* = - .008, *p* = 0.57). These results suggested that more memorable items tend to be perceived as tastier.


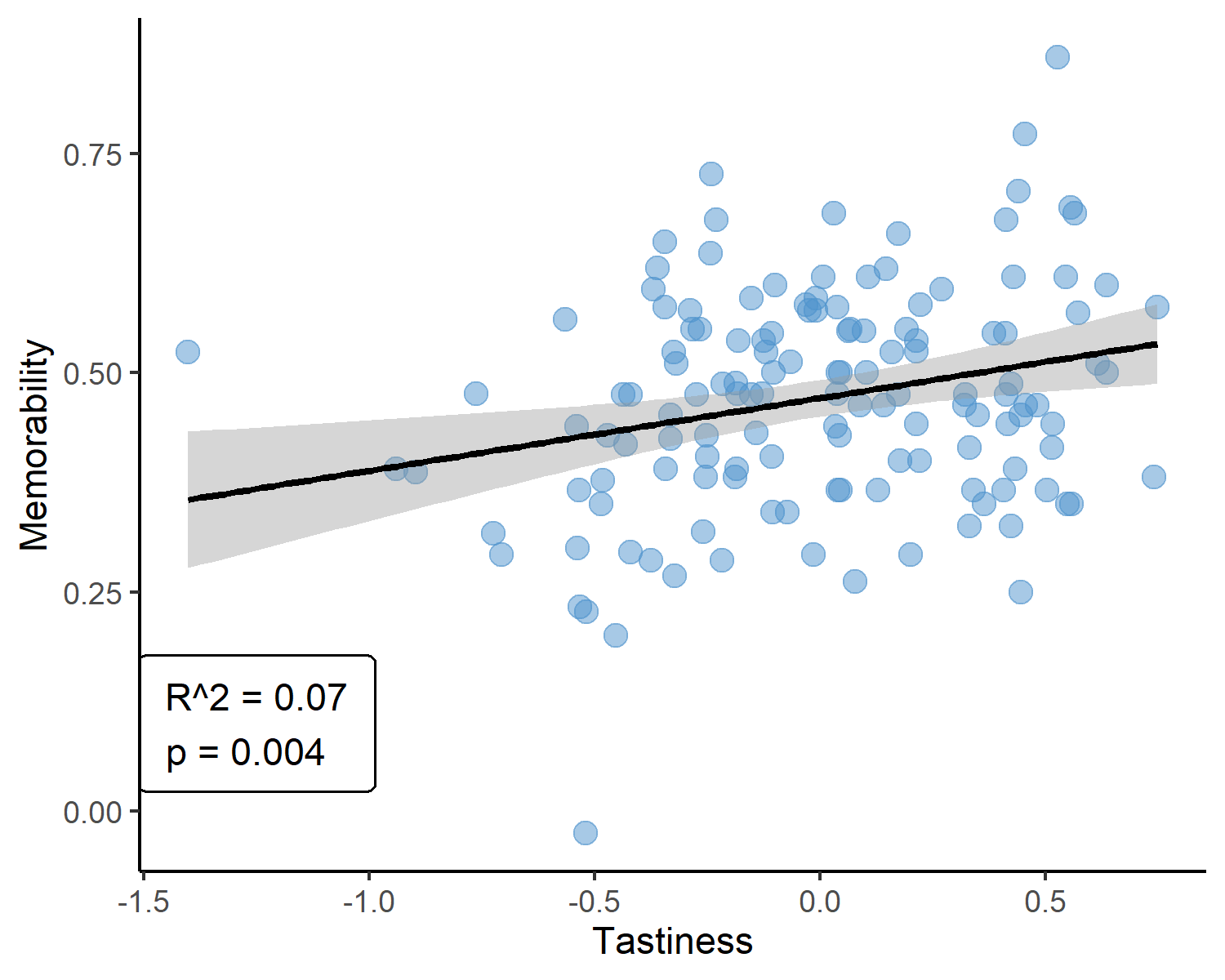


***Figure S4.*** Scatter plot for memorability scores and averaged tastiness score from the factor analysis on each food item. Each dot represents an individual food item. The solid black line is the predicted regression line, and the shaded area shows a 95% confidence interval.

***Expanded regressions and Bayesian Factors for Experiment 2C.*** To test the possible effect of memorability on the choice of food items, we applied the same expanded regressions that we did in Experiment 1B. However, we could not find any effect of word memorability on choices or RT in any form.

Same as Experiment 1B, we applied the Bayesian generalized regression models to test the null effects of |Δmem_word_| on choice and RT separately. For the Bayesian model of word memorability and choice preference, the estimate for the fixed effect was 0.01 (*Credible Intervals* = -0.08 – 0.10), and the calculated Bayes Factor in favor of the null was BF01 = $2.91\times{10}^{4}$. These results suggest that the data do not support the alternative hypothesis that |Δmem_word_| impacts participants’ choice preferences. For the Bayesian model testing the effect of |Δmem_word_| on RT, the estimate for the fixed effect was close to 0 (*Credible Intervals* = -0.02 – 0.01), and BF01 = $9.71\times{10}^{5}$. Similarly, these results show that the data is overwhelmingly in favor of the null hypothesis that |Δmem_word_| has no effect on RT.

**Supplementary Table**

| **Table S1** |  |
| --- | --- |
| *List of Words Used in Experiment 2.* | |
| **Image file name** | **Word(s)** |
| 1-milk.jpg | 1% milk |
| Air-popped-popcorn.jpg | popcorn |
| American-cheese.jpg | american cheese |
| Apple.jpg | apple |
| Avocado-green.jpg | avocado |
| Baby-Bell-cheese-withcrackers.jpg | babybel cheese with crackers |
| Bagel-cream-cheese.jpg | bagel with cream cheese |
| Bagel-plain.jpg | plain bagel |
| Baguette-with-olive-oil.jpg | baguette with olive oil |
| Baked-Potato.jpg | baked potato |
| Banana.jpg | banana |
| Black-Beans-with-rice.jpg | black beans with rice |
| Blueberries.jpg | blueberries |
| Broccoli-raw.jpg | broccoli |
| Broccoli-Cauliflower-raw.jpg | broccoli and cauliflower |
| Brownie.jpg | brownie |
| Burger-on-bun-homemade-80-lean.jpg | burger |
| Burrito-Amy-Bean-Cheese.jpg | burrito |
| Cantaloupe.jpg | cantaloupe |
| Carrot-Sticks.jpg | carrot sticks |
| Celery-Carrot-Sticks.jpg | celery and carrot sticks |
| Celery-sticks.jpg | celery sticks |
| Cheerios-dry.jpg | cheerios |
| Cheese-Nachos.jpg | cheese nachos |
| Cheese-cubes-mild-cheddar.jpg | cheese cubes |
| Cheeze-Itz.jpg | cheez-its |
| Cherries.jpg | cherries |
| Cherry-tomatoes.jpg | cherry tomatoes |
| Chicken-nuggets-w-BBQ-Sauce.jpg | chicken nuggets |
| Chicken-fingers-Perdue-Crispy-Chicken-Strips.jpg | chicken fingers |
| Chicken-noodle-soup-Campbells-Classic.jpg | chicken noodle soup |
| Chocolate-dark-covered-pretzels-Flipz.jpg | chocolate covered pretzels |
| Cocktail-shrimp.jpg | cocktail shrimp |
|  |  |
|  |  |
|  |  |
| **Image file name** | **Word(s)** |
| Choc-Chip-cookies-Entenmanns.jpg | chocolate chip cookies |
| Corn-on-Cob.jpg | corn on the cob |
| Craisins-Ocean-Spray.jpg | craisins |
| Croissant-plain.jpg | croissant |
| Cucumber-slices.jpg | cucumber |
| Cupcakes-mini-frosted.jpg | cupcakes |
| Doritos-Nacho-Cheese.jpg | doritos |
| Doughnuts-frosted-yeast-type.jpg | doughnuts |
| Edamame.jpg | edamame |
| Eggs-hard-boiled.jpg | hard boiled eggs |
| Egg-salad.jpg | egg salad |
| Falafel-withpita.jpg | falafel with pita |
| French-Fries.jpg | french fries |
| Eggs-fried.jpg | fried eggs |
| Fruit-Loops-w-whole-milk.jpg | froot loops with milk |
| Fruit-salad.jpg | fruit salad |
| Graham-crackers-Nabisco-Original.jpg | graham crackers |
| Granola-with-whole-milk.jpg | granola with milk |
| Granola-bar-Nature-Valley-Crunchy.jpg | granola bar |
| Grapefruit.jpg | grapefruit |
| Grapes.jpg | grapes |
| Green-beans-raw.jpg | green beans |
| Grilled-cheese.jpg | grilled cheese sandwich |
| Grilled-chicken-strips.jpg | grilled chicken strips |
| Hash-browns.jpg | hash browns |
| Hersheys-Kisses.jpg | hershey kisses |
| Hot-dog-on-bun-withmustard.jpg | hot dog |
| Ice-cream-sundae-ice-cream-whip-cream-cherry.jpg | ice cream sundae |
| Breyers-Vanilla-Ice-cream.jpg | vanilla ice cream |
| Kettle-Corn-Cracker-Jacks.jpg | kettle corn |
| Kit-Kat.jpg | kit kat |
| Kiwi.jpg | kiwi |
| Lettuce-salad.jpg | salad |
| Lollipops.jpg | lollipops |
| Lox.jpg | Lox |
|  |  |
| **Image file name** | **Word(s)** |
| Lucky-Charms-with-1-milk.jpg | lucky charms with milk |
| MM-candies.jpg | m&m's |
| Mac-and-cheese-Stouffers.jpg | mac and cheese |
| Mango.jpg | mango |
| Mashed-potatoes-withbutter.jpg | mashed potatoes |
| Melba-Toast-rye-Old-London.jpg | melba toast |
| Mini-muffins.jpg | mini muffins |
| Mozzarella-sticks-withmarinara-sauce.jpg | mozzarella sticks |
| Mushrooms-raw.jpg | mushrooms |
| Olives-green-withpimento.jpg | olives |
| Omelet-plain.jpg | omelet |
| Orange-sliced.jpg | orange |
| Oreos.jpg | oreos |
| Peanut-butter.jpg | peanut butter |
| Peach.jpg | peach |
| Peanut-Butter-Ritz-Bits-Crackers.jpg | peanut butter ritz crackers |
| Peanut-butter-1.5-oz-wt-withbanana-small.jpg | peanut butter with banana |
| Pear.jpg | pear |
| Pickles-dill.jpg | pickles |
| Pizza-restaurant.jpg | pizza |
| Popsicles.jpg | popsicles |
| Pop-Tart-strawberry-frosted.jpg | pop tarts |
| Potato-chips-Lays.jpg | potato chips |
| Pretzels.jpg | pretzels |
| Raisin-Bran-with-whole-milk.jpg | raisin bran with milk |
| Raisins.jpg | raisins |
| Raspberries.jpg | raspberries |
| Red-bell-pepper.jpg | bell pepper |
| Reeses-Peanut-butter-cups.jpg | reeses peanut butter cups |
| Reeses-Pieces.jpg | reeses pieces |
| Rice-Cakes.jpg | rice cakes |
| Rigatoni-plain.jpg | plain rigatoni |
| Rigatoni-withtomato-sauce.jpg | rigatoni and sauce |
| Ritz-crackers.jpg | ritz crackers |
| Roasted-potato.jpg | roasted potato |
| Salad-Spicy-Chicken-Caesar-Wendys-no-dressing.jpg | spicy chicken caesar salad |
| Salami.jpg | salami |
| Salmon-grilled.jpg | grilled salmon |
| Saltine-crackers.jpg | saltine crackers |
| Seaweed-dehydrated.jpg | seaweed |
| Shredded-wheat-1-biscuit-with-1-milk.jpg | shredded wheat with milk |
| Skim-milk.jpg | skim milk |
| Skittles.jpg | skittles |
| Minestrone-Soup-chunky-.jpg | minestrone soup |
| **Image file name** | **Word(s)** |
| Sour-patch-kids-candy.jpg | sour patch kids |
| Soy-crisps.jpg | soy crisps |
| Steak-T-bone.jpg | steak |
| Strawberries.jpg | strawberries |
| String-cheese.jpg | string cheese |
| Sun-Chips.jpg | sun chips |
| Sushi-with-condiments.jpg | sushi |
| Swedish-Fish.jpg | swedish fish |
| Sweet-Potato.jpg | sweet potato |
| Taco-beef-Taco-Bell-soft-supreme-.jpg | tacos |
| Trail-mix.jpg | trail mix |
| Chocolate-trail-mix.jpg | chocolate trail mix |
| Tribe-hummus-withpita-chips.jpg | hummus with pita chips |
| Triscuit.jpg | triscuit |
| Tuna-white-albacore-in-water-Bumble-Bee.jpg | tuna |
| Turkey-on-roll-with-lettuce-and-tomato.jpg | turkey sub |
| Turkey-breast-Oscar-Mayer-Deli-Select.jpg | turkey breast |
| Turkey-sandwich-roll-with-lettuce-and-tomato.jpg | turkey sandwich |
| Veggies-with-ranch-dip-Lays.jpg | veggies with ranch dip |
| Wheat-Bread-Pepperidge-Farm-100-WW.jpg | wheat bread |
| White-Bread-Pepperidge-Farm-Hearty-White.jpg | white bread |
| Whole-milk.jpg | whole milk |
| Yellow-popcorn.jpg | buttered popcorn |
| Rice-Beans.jpg | rice and beans |
| Yogurt-with-granola-strawberries.jpg | yogurt with granola & strawberries |
| Yogurt-pretzels.jpg | yogurt pretzels |
|  |  |
|  |  |
|  |  |
|  |  |
|  |  |
|  |  |
|  |  |
|  |  |
|  |  |
|  |  |
|  |  |
|  |  |
|  |  |
|  |  |

**Supplementary References**

1. Green, P. & MacLeod, C. J. SIMR : an R package for power analysis of generalized linear mixed models by simulation. *Methods Ecol Evol* 7, 493–498 (2016).
2. Chen, H., Cohen, P. & Chen, S. How Big is a Big Odds Ratio? Interpreting the Magnitudes of Odds Ratios in Epidemiological Studies. *Commun Statistics - Simul Comput* 39, 860–864 (2010).
3. Bürkner, P.-C. Advanced Bayesian Multilevel Modeling with the R Package brms. *R J* 10, 395 (2018).
4. Bürkner, P.-C. brms : An R Package for Bayesian Multilevel Models Using Stan. *J Stat Softw* 80, (2017).
5. Wiecki, T. V., Sofer, I. & Frank, M. J. HDDM: Hierarchical Bayesian estimation of the Drift-Diffusion Model in Python. *Front Neuroinform* 7, 14 (2013).
6. Needell, C. D. & Bainbridge, W. A. Embracing New Techniques in Deep Learning for Estimating Image Memorability. *Comput Brain Behav* 5, 168–184 (2022).
7. Lloyd, E. C. *et al.* Food Folio by Columbia Center for Eating Disorders: A Freely Available Food Image Database. *Frontiers in Psychology* 11, (2020).
8. Gorsuch, R. & Nelson, J. CNG scree test: an objective procedure for determining the number of factors. in *Proceedings of the annual meeting of the Society for Multivariate Experimental Psychology* (1981).
9. Raiche, G. An R package for parallel analysis and non graphical solutions to the Cattell scree test (R package version 2.3.3.1). https://CRAN.R-project.org/package=nFactors (2010).
